# Immunological profile and its clinical implications in pediatric infectious mononucleosis

**DOI:** 10.3389/fped.2025.1652825

**Published:** 2025-11-13

**Authors:** Jie Wang, Xinfeng Zhao, Cuiying Ye, Xudong Xu

**Affiliations:** Department of Clinical Laboratory, Hangzhou Children’s Hospital, Hangzhou, Zhejiang, China

**Keywords:** Epstein–Barr virus, infectious mononucleosis, granzyme, perforin, immune function

## Abstract

**Objective:**

To analyze the differences in peripheral blood immune function between children with infectious mononucleosis (IM) who developed liver injury (LI) and those non-liver injury (NLI), and to investigate the regulatory role of immune markers in IM infection.

**Methods:**

A total of 50 hospitalized children diagnosed with IM at Hangzhou Children's Hospital between November 2023 and August 2024 were enrolled as the Epstein–Barr Virus (EBV) infection group, including 19 without LI and 31 with LI. Additionally, 30 age-matched healthy children undergoing routine physical examinations during the same period were included as the control group. Flow cytometry was used to detect 12 cytokines, granzyme B (GzmB), perforin (PRF), regulatory T cells (Tregs), programmed cell death protein 1 (PD-1), and other immune markers in peripheral blood. CD100 levels were measured by ELISA.

**Results:**

The expression levels of IL-5, IFN-α, IL-2, IL-6, IL-10, IFN-γ, and IL-8 were significantly elevated in the EBV infection group compared to the control group (*P* < 0.01). Expression of GzmB and PRF in CD3^+^ and CD8^+^ T lymphocytes was significantly higher in the EBV group than in controls (*P* < 0.05). PD-1 and CD100 were also elevated in the EBV group (*P* < 0.01). Conversely, the expression of Tregs, CD28 in CD3^+^ and CD8^+^ T lymphocytes was significantly lower in the EBV group than in the control group (*P* < 0.01). Further comparison between the LI and non-liver injury (NLI) subgroups revealed that GzmB levels in CD3^+^ and CD8^+^ T lymphocytes were significantly higher in the LI group, while CD28 expression in CD3^+^ and CD8^+^ T lymphocytes was lower in the LI group compared to the NLI group (*P* < 0.05). Correlation analysis showed that PRF, PD-1 expression in CD3^+^ and CD8^+^ T lymphocytes, and IL-10 levels were positively correlated with EBV-DNA load (*P* < 0.05). GzmB in CD3^+^ and CD8^+^ T lymphocytes and IFN-*γ* levels were positively correlated with body temperature (*P* < 0.05). CD28 expression in CD3^+^ and CD8^+^ T lymphocytes was negatively correlated with ALT levels (*P* < 0.05).

**Conclusion:**

EBV infection-induced IM is associated with abnormal expression of various immune markers in peripheral blood.The high expression of GzmB and low expression of CD28 are associated with LI in IM.

## 1 Introduction

Epstein–Barr virus (EBV) is a relatively common double-stranded DNA virus belonging to the gamma subfamily of herpesviruses. It can be transmitted through saliva (commonly known as the “kissing disease”), blood transfusion, organ transplantation, and other routes, and is associated with a variety of clinical diseases (1). EBV exhibits widespread susceptibility in the general population. EBV primary infection of children can result in infectious mononucleosis (IM), an acute serious condition characterized by massive lymphocytosis.Previous studies have shown that the global seropositivity rate of EBV-induced IM exceeds 90%, with a steadily increasing incidence among children (2). The typical clinical manifestations of IM include fever, pharyngitis, and lymphadenopathy. Pediatric IM often involves multiple organ systems and is frequently accompanied by varying degrees of liver injury (LI). EBV-induced liver damage is primarily mediated through cellular immune responses and inflammation (3). Therefore, this study aims to evaluate immune parameters such as granzyme B (GzmB), perforin (PRF), regulatory T cells (Tregs), programmed cell death protein 1 (PD-1), CD100, and cytokine profiles in children with EBV infection, using flow cytometry and ELISA. By comparing differences in peripheral blood immune function among children with IM, we seek to explore the role of these immune indicators in the development of LI and provide novel insights for clinical diagnosis and treatment.

## 2 Materials and methods

### 2.1 Study design and participants

A total of 50 children diagnosed with IM and hospitalized at Hangzhou Children's Hospital between November 2023 and August 2024 were included in the analysis. Among them, 22 were male and 28 were female, with a mean age of (5.55 ± 2.95) years (range: 8 months to 14 years). Of the 50 patients, 31 had LI and 19 did not. Other viral hepatitis infections were excluded in children diagnosed with LI. Inclusion criteria: Diagnosis met the criteria outlined in the Expert Consensus on the Diagnosis and Treatment of Epstein–Barr Virus–Associated Diseases in Children (4). The criteria of LI: Liver damage was diagnosed if the biochemical analyzer showed abnormal values of serum aspartate aminotransferase (AST), alanine aminotransferase (ALT),gamma-Glutamyl Transferase(GGT), and/or alkaline phosphatase (ALP).Exclusion criteria: Congenital immunodeficiency or comorbid immune system disorders; a history of allergic diseases; Concomitant malignancies or hematologic disorders; Use of glucocorticoids, cytotoxic agents, or other immunosuppressive drugs within 3 months prior to enrollment. All 50 children were cured and discharged, and they will be followed up after discharge to observe whether any chronic conditions develop. An additional 30 healthy children undergoing routine physical examinations during the same period were recruited as the control group. There were no statistically significant differences in age or sex between the two groups (*P* > 0.05), indicating comparability. In addition, the children in the control group tested negative for Epstein–Barr virus nucleic acids and antibodies, and they had no history of liver, kidney, gastrointestinal, metabolic abnormalities, or neurological diseases. This study was conducted in compliance with the Declaration of Helsinki and approved by the Hangzhou Children's Hospital Clinical Research Ethics Committee (Protocol Number: No. 2023-IRB-74). Clinical data for both groups are shown in Table 1.

**Table 1:** Comparison of clinical general conditions between EBV infection group and control group.

Clinical characteristics	EBV-infected LI group	EBV-infected NLI group	Control group
Number of cases	31	19	30
Age (year)	6.52 ± 2.99	3.98 ± 2.13	5.57 ± 2.91
Gender (male/female)	12/19	10/9	14/16
Fever [*n* (%)]	31 (100%)	19 (100%)	–
Pharyngitis [*n* (%)]	31 (100%)	19 (100%)	–
Lymphadenopathy [*n* (%)]	31 (100%)	19 (100%)	–
Hepatomegaly [*n* (%)]	7 (22.6%)	1 (5.3%)	–
Splenomegaly [*n* (%)]	19 (61.3%)	9 (47.4%)	–
Palpebral edema [*n* (%)]	19 (61.3%)	12 (63.2%)	–
EBV-DNA[copies/ml]	500 (500,2000)	700 (500,2000)	Negative
ALT(U/L)	90.00 (53.00, 163.00)	23.0 (15.00, 24.00)	12.00 (11.00, 15.50)
AST(U/L)	88.00 (56.00, 123.00)	38.00 (32.00, 41.00)	28.00 (24.75, 32.00)
GGT(U/L)	40.00 (27.00, 110.00)	17.00 (15.00, 19.00)	16.00 (15.00, 19.00)
ADA(U/L)	57.00 (48.00, 78.00)	41.00 (33.00, 47.00)	14.00 (12.00, 16.00)

### 2.2 Reagents and instruments

Flow cytometry reagents (Qingdao Raisecare Biotech Co. Ltd.), ELISA kits (Shanghai Jianglai Biotechnology Co. Ltd.), Biochemistry reagents (Ningbo Purebio Co., Ltd.), Hematology analyzers and reagents (Sysmex Corporation, Japan), CRP detection instruments and reagents (Shanghai Upper Co. Ltd.), Flow cytometer (Qingdao Raisecare Biotech Co. Ltd.), Automatic biochemical analyzer (Hitachi 7600, Japan).

### 2.3 Research methods

#### 2.3.1 Sample collection and processing

For IM patients, peripheral venous blood was collected within 24 h of admission; for healthy controls, fasting peripheral venous blood was collected in the morning. Two types of blood samples were obtained: 3 mL of non-anticoagulated blood and 3 mL of EDTA-anticoagulated blood. The non-anticoagulated blood was centrifuged at 3,000 rpm for 10 min, and the serum was stored at −80 °C. EDTA-anticoagulated blood was used immediately for immune marker detection.

#### 2.3.2 Cytokine detection

Twelve cytokines (IL-5, IFN-α, IL-2, IL-6, IL-1β, IL-10, IFN-*γ*, IL-8, IL-17, IL-4, IL-12p70, and TNF-α) in serum were detected using multiplex microsphere-based flow immunofluorescence. The procedure was performed strictly according to the manufacturer's instructions, and the assay was run on a Beckman Coulter flow cytometer (USA).

#### 2.3.3 Detection of GzmB and PRF

Flow cytometry was used for detection. After gentle inversion (10 times) of EDTA-anticoagulated whole blood, 50 μl was transferred into flow cytometry tubes. Surface antibodies (CD45, CD3, CD8, CD16, CD56; 5 μl each) were added, followed by 120 μl fixative and incubation in the dark for 15 min. Then, 2 mL of 1× permeabilization solution was added and incubated in the dark for 15 min. Samples were centrifuged at 200–300 g for 5 min, supernatant discarded, and intracellular antibodies for GzmB and PRF (5 μl each) were added. After vortexing and 15 min dark incubation, 200 μl of 1× PBS buffer was added. Samples were vortexed and analyzed by flow cytometry.

#### 2.3.4 Detection of tregs, PD-1, and other immune markers

Following gentle inversion of EDTA-anticoagulated blood, 5 μl each of fluorescent-labeled monoclonal antibodies against CD3, CD4, CD25, and CD127 were added to the bottom of the flow cytometry tube. Additionally, 25 μl of CD45-PECy7, CD3-PerCP, CD4-APC, CD8-APC-Cy7, PD-1-PE, IgG1-PE (isotype control), CD28-FITC, and IgG1-FITC (isotype control) were added. After vortexing and 15 min dark incubation, 450 μl of 1× red blood cell lysis buffer was added for 15 min hemolysis in the dark at room temperature. Samples were then analyzed by flow cytometry.

#### 2.3.5 CD100 detection

CD100 was measured using a commercial ELISA kit according to the manufacturer's instructions. After preparing the working solution, 100 μl of sample or standard was added per well and incubated at 37 °C for 60 min. Then, 100 μl of biotinylated antibody was added and incubated for another 60 min. After washing, 100 μl of enzyme conjugate was added and incubated for 30 min. After another wash, 90 μl of substrate was added, followed by stop solution. The absorbance was measured using a microplate reader.

### 2.4 Statistical methods

Statistical analyses were performed using SPSS 20.0 software. Data were tested for normality. For normally distributed variables, results were expressed as mean ± standard deviation, and t-tests were used for group comparisons. For non-normally distributed data, the median (interquartile range) [M(P25–P75)] was used, and group comparisons were made using the Mann–Whitney *U* test or Kruskal–Wallis H test. Pearson correlation analysis was conducted for correlation evaluation. A *P*-value < 0.05 was considered statistically significant.

## 3 Results

### 3.1 Clinical characteristics of children with EBV infection

A total of 50 pediatric patients with IM caused by EBV infection were enrolled in this study. Among them, 19 cases did not present with LI (Non-Liver Injury, NLI), while 31 cases exhibited LI. All patients presented with classical clinical manifestations, including fever, pharyngitis, and lymphadenopathy, accounting for 100% of the cohort. The average body temperature among febrile patients was (38.70 ± 0.76)°C. All patients tested positive for EBV DNA. Abdominal ultrasound revealed hepatomegaly in 8 cases (16.0%) and splenomegaly in 28 cases (56.0%). Compared to NLI patients, those in the LI group showed significantly elevated levels of alanine aminotransferase (ALT, *P* < 0.001), aspartate aminotransferase (AST, *P* < 0.001), and gamma-glutamyl transferase (GGT, *P* < 0.001). No significant difference in EBV-DNA content was observed in the two groups (*P* = 0.467).All patients were successfully discharged after treatment. The baseline clinical characteristics of the enrolled children are summarized in Table 1.

### 3.2 Comparison of cytokine profiles between EBV infection group and healthy controls

We assessed the concentrations of 12 cytokines (IL-5, IFN-α, IL-2, IL-6, IL-1β, IL-10, IFN-γ, IL-8, IL-17, IL-4, IL-12p70, and TNF-α) in peripheral blood from the EBV infection group and healthy control group. Compared with the control group, the EBV-infected children exhibited significantly elevated levels of IL-5, IFN-α, IL-2, IL-6, IL-10, IFN-γ, and IL-8 (all *P* < 0.01) (Table 2). However, no statistically significant differences in cytokine levels were observed between the LI and NLI subgroups within the EBV infection group.

**Table 2:** Comparison of cytokine profiles between EBV-infected and control groups [M (P25–P75)] (%).

Test items (pg/mL)	EBV-infected group (*n* = 50)	Control group (*n* = 30)	Statistical value	*P*
IL-5	4.92 (3.36, 7.46)	2.72 (2.00, 4.36)	−3.414	0.001
IFN-α	1.85 (1.20, 2.71)	0.98 (0.75, 1.66)	−3.493	0.000
IL-2	1.57 (1.19, 2.05)	0.94 (0.82, 1.13)	−4.259	0.000
IL-6	16.88 (8.88, 37.19)	2.17 (1.26, 3.16)	−6.529	0.000
IL-1β	4.58 (2.84, 7.56)	3.12 (1.66, 7.50)	−1.461	0.144
IL-10	32.46 (15.25, 55.87)	1.66 (1.06, 2.52)	−6.748	0.000
IFN-γ	99.97 (37.69, 179.80)	4.84 (3.48, 6.45)	−6.688	0.000
IL-8	36.47 (18.36, 82.03)	20.44 (7.89, 39.50)	−2.793	0.005
IL-17	3.11 (2.52, 4.57)	2.86 (1.96, 3.36)	−1.605	0.108
IL-4	0.67 (0.55, 1.00)	0.65 (0.52, 0.95)	−0.333	0.739
IL-12P70	0.75 (0.52, 1.28)	0.63 (0.42, 0.95)	−1.565	0.118
TNF-α	5.17 (3.08, 10.16)	4.46 (2.62, 8.30)	−1.039	0.299

### 3.3 Comparison of immune indicators including GzmB, PRF, tregs, PD-1, and CD100 between EBV infection group and healthy controls

**Figure 1:**
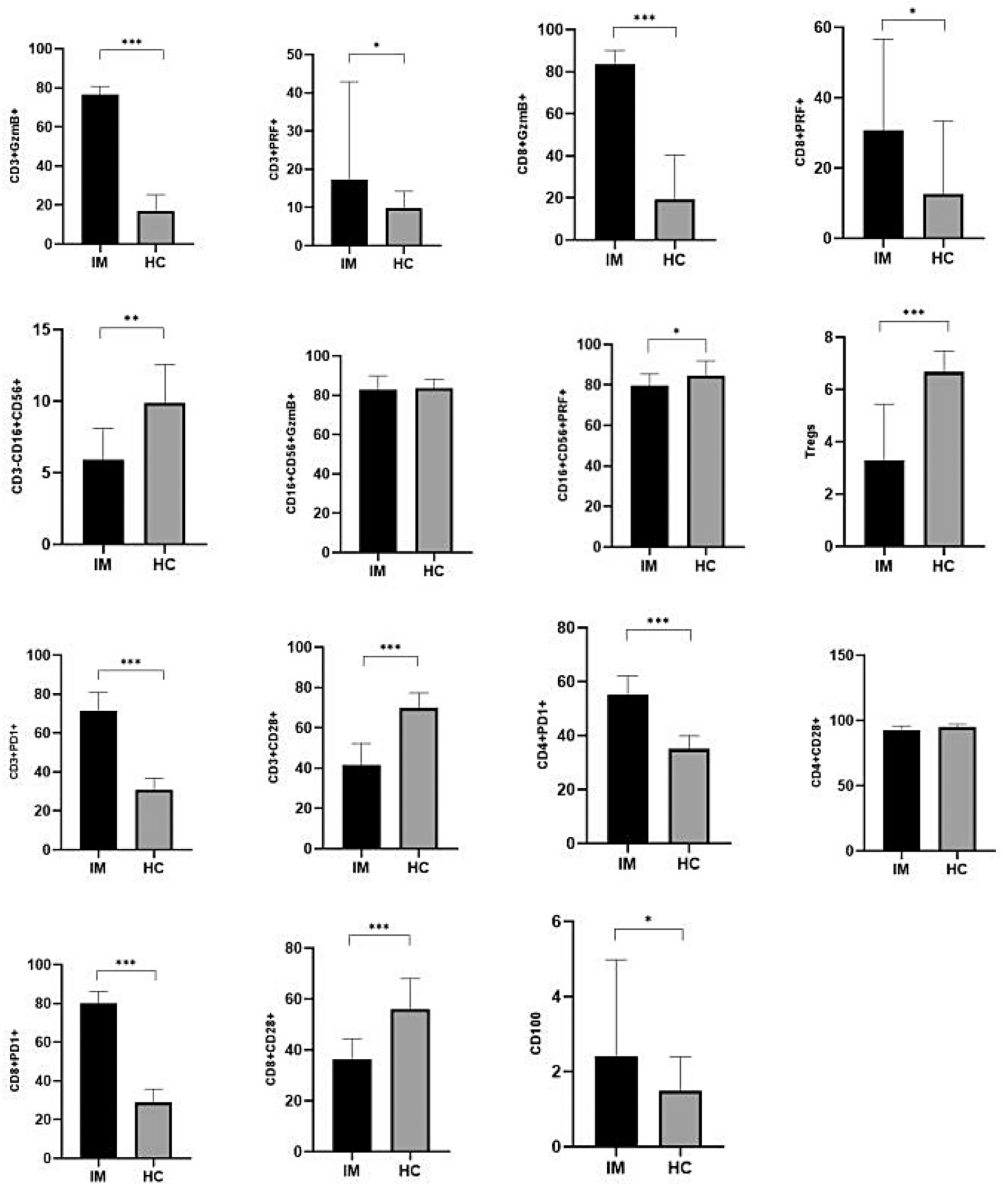
Comparison of immune-related indicators between EBV-infected group and control group [M (P5-P75)](%). *P < 0.05; **P < 0.01; ***P < 0.001.

The results of this study demonstrated that the expression levels of GzmB and PRF in CD3^+^ T lymphocytes were significantly elevated in the EBV-infected group compared to the control group (*P* < 0.05). Similarly, GzmB and PRF expression in CD8^+^ T lymphocytes was significantly higher in the EBV-infected children than in control group (*P* < 0.05). Furthermore, the proportions of PD-1 among CD3^+^, CD4^+^, and CD8^+^ T lymphocytes were markedly increased in the EBV-infected group compared to the control group, with statistically significant differences (*P* < 0.01). Expression of CD100 was also significantly elevated in the EBV-infected group (*P* < 0.05). In contrast, the frequencies of Tregs, CD28 among CD3^+^ and CD8^+^ T lymphocytes were significantly lower in the EBV-infected group compared to controls (*P* < 0.01). These findings are summarized in Table 3; Figure 1.

**Table 3:** Comparison of immune-related indicators between EBV-infected group and control group [M (P25-P75)] (%).

Test items	EBV-infected group (*n* = 50)	Control group (*n* = 30)	Statistical value	*P*
CD3 + GzmB+	76.67 (57.55, 80.72)	16.93 (9.26, 25.42)	−5.923	0.000
CD3 + PRF+	17.31 (7.32, 42.90)	9.83 (5.40, 14.21)	−2.335	0.020
CD8 + GzmB+	83.71 (71.27, 90.16)	19.38 (7.32, 40.37)	−5.635	0.000
CD8 + PRF+	30.83 (9.85, 56.68)	12.75 (4.98, 33.35)	−2.554	0.011
CD3-CD16 + CD56+	5.92 (4.47, 8.09)	9,89 (6.42, 12.55)	−3.354	0.001
CD16 + CD56 + GzmB+	82.95 (74.18, 89.86)	83.59 (76.47, 88.21)	−0.184	0.851
CD16 + CD56 + PRF+	79.57 (66.40, 85.54)	84.67 (76.21, 91.87)	−2.385	0.017
Tregs	3.30 (2.32, 5.44)	6.68 (5.49, 7.47)	−4.641	0.000
CD3 + PD1+	71.31 (62.82, 80.88)	30.74 (22.45, 36.69)	−6.172	0.000
CD3 + CD28+	41.56 (32.71, 52.18)	69.85 (63.95, 77.29)	−6.042	0.000
CD4 + PD1+	55.28 (46.63, 62.11)	35.01 (26.73, 39.89)	−5.496	0.000
CD4 + CD28+	92.30 (87.66, 95.63)	94.89 (88.13, 97.24)	−1.401	0.161
CD8 + PD1+	80.22 (66.92, 86.16)	28.95 (17.47, 35.83)	−6.857	0.000
CD8 + CD28+	36.33 (26.27, 44.38)	55.98 (44.44, 68.05)	−4.711	0.000
CD100	2.41 (1.27, 4.98)	1.50 (0.82, 2.40)	−2.281	0.023

### 3.4 Comparison of immune markers including GzmB, PRF, tregs, PD-1, and CD100 between EBV-infected children with and without LI

**Figure 2:**
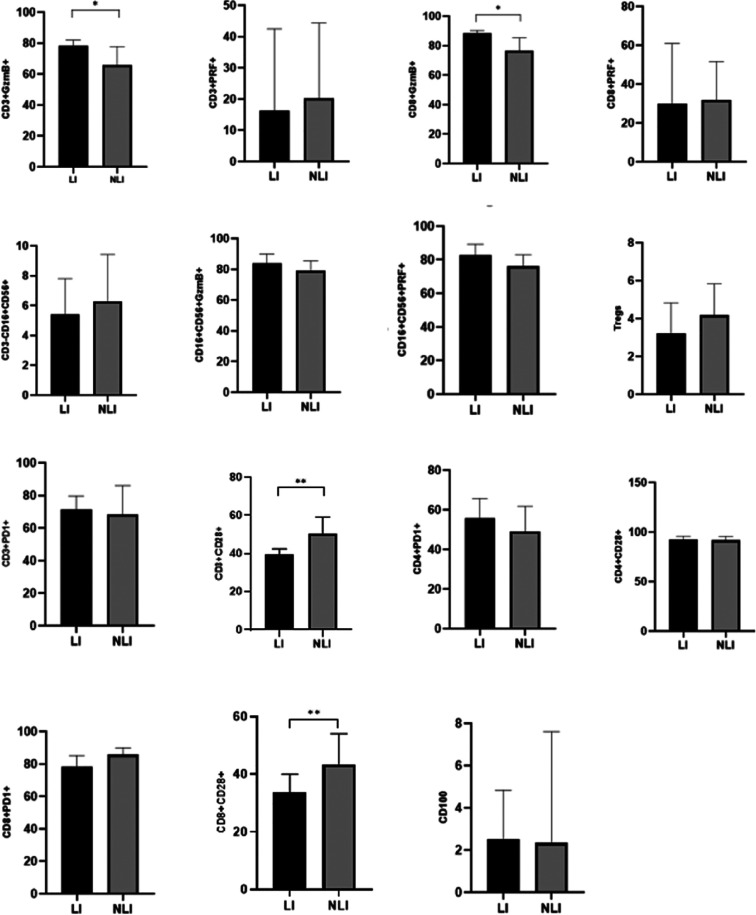
Comparison of immune-related indicators between EBV-infected LI and NLI group [M (P25-P75)](%). *P < 0.05; **P < 0.01; ***P < 0.001.

A further comparison was conducted between the LI group and the NLI group among EBV-infected children. The results showed that the expression levels of GzmB in both CD3^+^ T lymphocytes and CD8^+^ T lymphocytes were significantly higher in the LI group compared to the NLI group, with statistically significant differences (*P* < 0.05). In contrast, the expression of CD28 on both CD3^+^ T lymphocytes and CD8^+^ T lymphocytes was significantly lower in the LI group than in the NLI group (*P* < 0.01), as shown in Table 4; Figure 2.

**Table 4:** Immune-related comparison between EBV-infected LI and NLI group [M (P25–P75)] (%).

Test items	EBV-infected LI group (*n* = 31)	EBV-infected NLI group (*n* = 19)	Statistical value	*P*
CD3 + GzmB+	78.21 (62.10, 82.04)	65.88 (37.39, 77.80)	−2.329	0.020
CD3 + PRF+	16.29 (7.82, 42.42)	20.33 (5.04, 44.34)	−0.010	0.992
CD8 + GzmB+	88.52 (77.74, 90.34)	76.71 (51.35, 85.23)	−2.508	0.012
CD8 + PRF+	29.74 (15.70, 60.90)	31.91 (4.61, 51.55)	−0.989	0.322
CD3-CD16 + CD56+	5.40 (4.17, 7.80)	6.28 (5.36, 9.43)	−1.749	0.080
CD16 + CD56 + GzmB+	83.82 (75.22, 89.92)	79.11 (69.92, 85.36)	−1.769	0.077
CD16 + CD56 + PRF+	82.61 (67.26, 89.19)	76.19 (60.76, 82.93)	−1.479	0.139
Tregs	3.22 (2.16,4.82)	4.17 (2.45,5.83)	−1.269	0.204
CD3 + PD1+	71.48 (65.27,79.69)	68.29 (54.03,86.01)	−0.300	0.764
CD3 + CD28+	39.26 (30.83,42.42)	50.17 (40.61,59.19)	−3.058	0.002
CD4 + PD1+	55.77 (48.93,65.68)	49.05 (39.82,61.75)	−1.319	0.187
CD4 + CD28+	92.65 (89.38,95.76)	91.76 (82.45,95.59)	−1.099	0.272
CD8 + PD1+	78.46 (67.53,85.14)	85.85 (63.10,90.02)	−0.199	0.230
CD8 + CD28+	33.76 (22.60,40.03)	43.48 (32.70,54.09)	−2.998	0.003
CD100	2.52 (1.40,4.84)	2.33 (0.72,7.61)	−0.130	0.897

### 3.5 Correlation analysis

Correlation analysis between immune indicators and EBV-DNA levels in EBV-infected children revealed that the expression of PRF, PD-1 in CD3^+^ and CD8^+^ T lymphocytes and IL-10 were positively correlated with EBV-DNA load (*r* = 0.288, *P* = 0.042; *r* = 0.335, *P* = 0.017; *r* = 0.336, *P* = 0.017; *r* = 0.294, *P* = 0.039; *r* = 0.334, *P* = 0.018). In contrast, IL-12p70 was negatively correlated with EBV-DNA load (*r* = −0.302, *P* = 0.033). Further analysis of immune indicators and clinical parameters in EBV-infected children showed that GzmB in CD3^+^ and CD8^+^ T lymphocytes, as well as IFN-*γ*, were positively correlated with body temperature (*r* = 0.285, *P* = 0.044; *r* = 0.293, *P* = 0.039; *r* = 0.344, *P* = 0.014). Correlation analysis between immune and biochemical indicators revealed that CD28 expression in CD3^+^ and CD8^+^ T lymphocytes was negatively correlated with ALT levels (*r* = −0.439, *P* = 0.001; *r* = −0.364, *P* = 0.009), as shown in Table 5. No correlation has been found so far between immunological indicators and other biochemical indicators.

**Table 5:** Results of correlation analysis of immune indicators of EBV infection.

Indicators	EBV-DNA load		Body temperature		ALT	
	*r*	*P*	*r*	*P*	*r*	*P*
CD3 + GzmB+	0.068	0.640	0.285	0.044*	0.268	0.060
CD3 + PRF+	0.288	0.042*	0.047	0.743	−0.056	0.699
CD8 + GzmB+	0.237	0.098	0.293	0.039*	0.251	0.079
CD8 + PRF+	0.335	0.017*	0.024	0.868	−0.073	0.612
CD3-CD16 + CD56+	0.203	0.156	−0.046	0.751	−0.263	0.065
CD16 + CD56 + GzmB+	0.077	0.596	0.145	0.316	0.076	0.599
CD16 + CD56 + PRF+	0.099	0.494	0.091	0.528	−0.034	0.815
Tregs	−0.033	0.818	−0.103	0.476	−0.182	0.206
CD3 + PD1+	0.336	0.017*	−0.213	0.137	0.123	0.396
CD3 + CD28+	−0.085	0.556	0.061	0.672	−0.439	0.001*
CD4 + PD1+	0.199	0.165	0.043	0.769	0.274	0.050
CD4 + CD28+	0.103	0.478	0.058	0.690	0.104	0.471
CD8 + PD1+	0.294	0.039*	−0.268	0.059	0.053	0.715
CD8 + CD28+	0.049	0.736	−0.038	0.794	−0.364	0.009*
CD100	−0.274	0.054	−0.007	0.964	0.089	0.540
IL-5	0.095	0.513	−0.173	0.230	0.028	0.849
IFN-α	0.035	0.809	0.218	0.129	−0.116	0.421
IL-2	0.099	0.493	−0.064	0.661	−0.037	0.801
IL-6	0.105	0.470	0.254	0.075	−0.104	0.474
IL-1β	0.081	0.578	−0.007	0.962	0.117	0.419
IL-10	0.334	0.018*	0.242	0.091	−0.121	0.404
IFN-γ	0.142	0.325	0.344	0.014*	0.002	0.988
IL-8	−0.124	0.393	0.039	0.786	0.098	0.497
IL-17	−0.016	0.915	−0.155	0.282	0.135	0.351
IL-4	0.149	0.301	−0.112	0.439	0.078	0.588
IL-12P70	−0.302	0.033*	0.011	0.940	0.057	0.696
TNF-α	0.107	0.458	−0.009	0.950	−0.051	0.725

* *P* < 0.05.

## 4 Discussion

EBV infection is relatively common among preschool-aged children and is prone to causing various acute infectious diseases, with IM being one of the most prevalent. Although IM is generally a self-limiting disease with a favorable prognosis, the immature immune system in children can, in rare cases, result in complications such as peripheral neuritis, meningitis, or LI. The outcome and prognosis of IM caused by EBV infection may vary significantly depending on the host's immune status (5). Most children with IM experience transient elevations in liver enzymes, which are typically self-limited and rarely progress to chronic conditions. However, in cases of chronic active EBV infection, EBV-related LI can follow an irreversible chronic course, potentially leading to liver cirrhosis (6). In this study, the primary clinical manifestations of IM included fever, eyelid edema, lymphadenopathy, hepatosplenomegaly, and pharyngitis. LI was mainly characterized by elevated ALT and AST levels, with some cases also showing increased GGT and ADA levels, consistent with findings from previous reports (7, 8).In addition, this study found that the children in the liver injury group were older, suggesting that the probability and severity of IM liver injury were associated with age, which is consistent with related studies (9).

The immune system and inflammatory responses in children with IM are closely related to the disease's onset and progression. Cytokines, including interleukins, chemokines, interferons, and tumor necrosis factors, play key roles in immune regulation. Disruption of cytokine balance can lead to pathological changes in the host. Previous studies have confirmed that IL-17A, IL-22, Tim-3, and Galectin-9 are involved in the immunopathogenesis of EBV-induced IM in children, suggesting a possible association with immune-mediated LI (10). In our study, analysis of 12 cytokines revealed significantly elevated levels of several cytokines (IL-5, IFN-α, IL-2, IL-6, IL-10, IFN-γ, and IL-8) in the EBV-infected group, indicating the activation of cytokines following EBV infection. This finding is consistent with previous reports (11). However, no significant differences in cytokine levels were observed between the LI and NLI subgroups.

Studies have shown that cytotoxicity mediated by granzyme and PRF plays an essential role in controlling infections (12). For example, research by Sutton VR et al. demonstrated that increased expression of GzmB and PRF in peripheral blood lymphocytes contributes to viral clearance in HBV infection (13). In our study of 50 children with IM, we observed elevated expression of GzmB and PRF in peripheral blood CD3^+^ and CD8^+^ T lymphocytes of the EBV-infected group. Furthermore, the levels of GzmB in CD3^+^ and CD8^+^ T cells were significantly higher in the LI subgroup, suggesting enhanced direct cytotoxic activity in children with LI due to EBV infection. This cytotoxic process may contribute to EBV-induced liver damage. Therefore, the high expression of GzmB and PRF in CD3^+^ and CD8^+^ T lymphocytes may play a crucial role in the pathogenesis of LI in IM. Upon reviewing the cases, some children presented with atypical symptoms, making it difficult to determine the exact onset date. Therefore, the specific time interval from symptom onset to sample collection could not be established. As a result, this study collected samples 24 h after hospitalization to measure liver enzymes and related immune markers, which is one of the limitations of this study.

In addition, various immune mechanisms play vital roles in viral infections, such as Tregs and PD-1 (14). Previous studies have reported an imbalance between Tregs and T helper 17 cells (Tregs/Th17) in children with EBV infection. During the EBV clearance process, Th17 cells exhibit increased proliferation, leading to an amplified inflammatory cascade response (15). PD-1, a member of the CD28 superfamily, is a crucial component of the CD28/CTLA-4/ICOS costimulatory receptor family and plays a key role in negatively regulating T and B cell immune responses. Persistent exposure to viral antigens can result in elevated PD-1 expression and immune cell exhaustion (16). Some studies have found through the transcriptome of EBV and single cell sequenced TM cells that EBV is abundant in functional Tem cells, CD28 can be used as continuous indicators to interrogate the antiviral ability of T cells (17).

In chronic diseases, compared to EBV^−^DLBCL, EBV^+^ DLBCL showed that both CD4^+^ and CD8^+^ T cells demonstrated elevated PD-1 expression.There was also a significant decline in CD28^+^ KLRG1^−^and CD28^+^ CD57^−^KLRG1^−^subsets among CD8^+^ T cells in EBV+ patients (18).

In our study, the expression of PD-1 on CD3^+^, CD4^+^, and CD8^+^ T lymphocytes was significantly increased in children with EBV infection, whereas the expression levels of Tregs, as well as CD28 within CD3^+^ and CD8^+^ T lymphocytes, were markedly decreased, indicating a state of immune imbalance, consistent with the results reported by relevant (19). In our study, we found an imbalance in Tregs; however, unfortunately, Th17 was not measured in this study, so we could not explore the impact of Th17/Treg imbalance on Epstein–Barr virus infection, this is a limitation of this study. Related studies show an important role for CD28 in the priming of naïve EBVspecific CD8^+^ *T* cell responses (20).Some studies believe that decreased CD28 expression could also indicate a shift from naive CD8^+^ cells to effector memory *T* cells (21). CD100, a member of the semaphorin family of immune signaling molecules, exists in both soluble (sCD100) and membrane-bound (mCD100) forms and is also known as Sema4D. It plays important physiological roles in immune regulation, such as enhancing antigen-specific responses and functioning as a costimulatory molecule on T cells. CD100 is also implicated in the pathogenesis of various diseases, including cardiovascular conditions and HIV infection (22, 23). Elevated levels of Sema4D have been observed in patients with liver cirrhosis and are positively correlated with liver and gallbladder damage, suggesting a role in inflammation-associated hepatic injury (24). Our study demonstrated that CD100 expression was significantly elevated in children with EBV-induced IM, indicating that increased CD100 may play a role in the immune dysregulation associated with EBV infection. However, in this study, no significant difference in CD100 was found between the LI and NLI groups. It remains unclear whether CD100 plays a role in liver injury following EB virus infection. Previous studies have shown that in chronic viral infections and malignancies, sCD100 levels are reduced while mCD100 expression on CD8^+^ *T* cells is elevated—an imbalance potentially linked to CD8^+^ *T* cell functional exhaustion (25).

Previous studies have reported that in EBV infection, IL-17A and IL-22 are positively correlated with CD3^+^ and CD3^+^ CD8^+^ T lymphocytes, while Tim-3 and Gal-9 show negative correlations with these cell populations. These findings suggest that Tim-3 and Gal-9 binding may contribute to CD8^+^ *T* cell dysfunction during persistent viral infections (10). Zhang YH et al. found that elevated liver enzymes in children with EBV infection were closely associated with CD8^+^ *T* cells (26). CD8^+^ *T* cells infected with EBV may bind to specific adhesion molecules expressed by hepatic Kupffer cells and mediate cytotoxic effects or induce apoptosis of target cells through Fas/FasL and PRF/granzyme pathways (27). In our study, our correlation analysis revealed a positive relationship between EBV-DNA viral load and the expression levels of IL-10 and PRF, PD-1 in CD3^+^ and CD8^+^ T lymphocytes.Body temperature was positively correlated with IFN-γ and GzmB levels in CD3^+^ and CD8^+^ T lymphocytes.Increased ALT levels were positively correlated with CD28 expression in CD3^+^ and CD8^+^ T lymphocytes. The above results suggest that there is a certain correlation between immune indicators and clinical conditions. However, this study also has some limitations. First, this study primarily collected data from hospitalized children and did not include outpatient cases. Second, the sample size was relatively small. Third, the results of this study are based on a single-center study of the children. In future research, we hope to expand the sample size, include more diverse types of patients, and use more refined detection methods to explore these mechanisms further.

In summary, EBV-induced infectious mononucleosis is characterized by dysregulated expression of multiple immune markers in peripheral blood.The high expression of GzmB and low expression of CD28 are associated with LI in IM, and further mechanistic exploration is warranted.

## Data Availability

The original contributions presented in the study are included in the article/Supplementary Material, further inquiries can be directed to the corresponding author.
