# Supplementary material for: Immunological profile and its clinical implications in pediatric infectious mononucleosis
Source: Front Pediatr. 2025 Nov 13;13:1652825. doi: 10.3389/fped.2025.1652825 (PMC12657469; doi:10.3389/fped.2025.1652825)
Supplement: Supplementary file 1 [file Supplementaryfile1.docx]

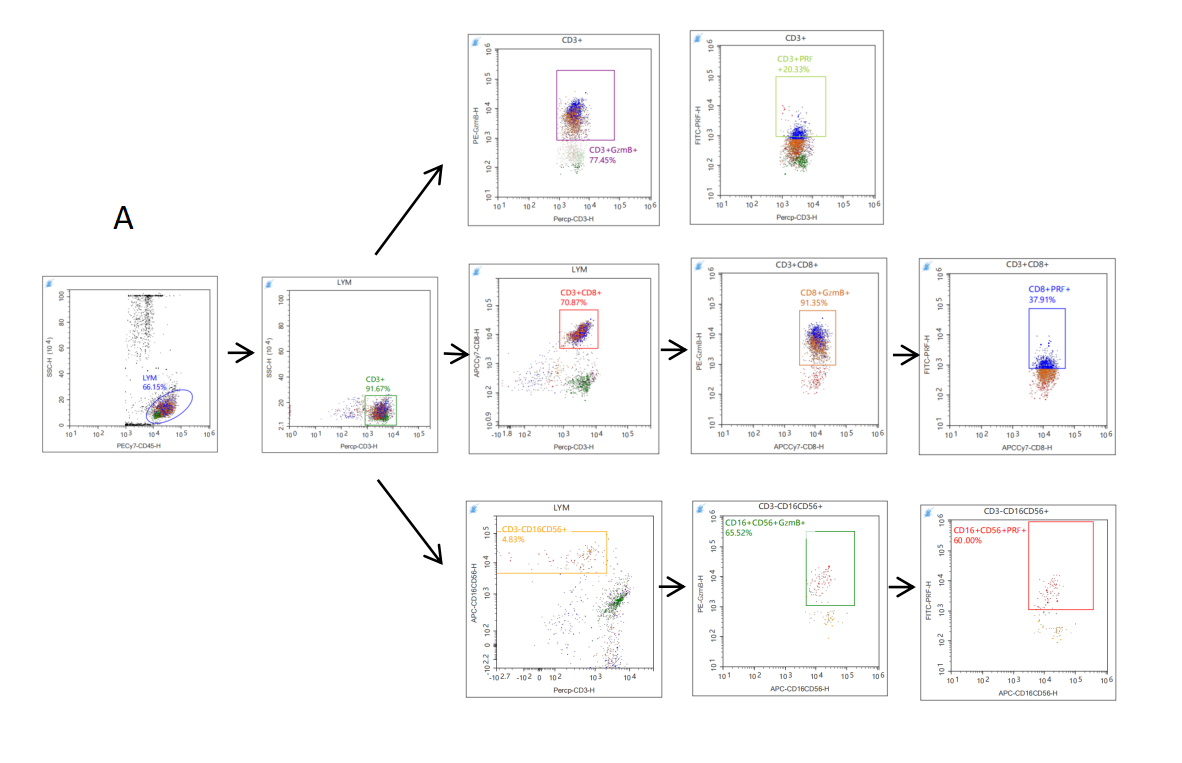


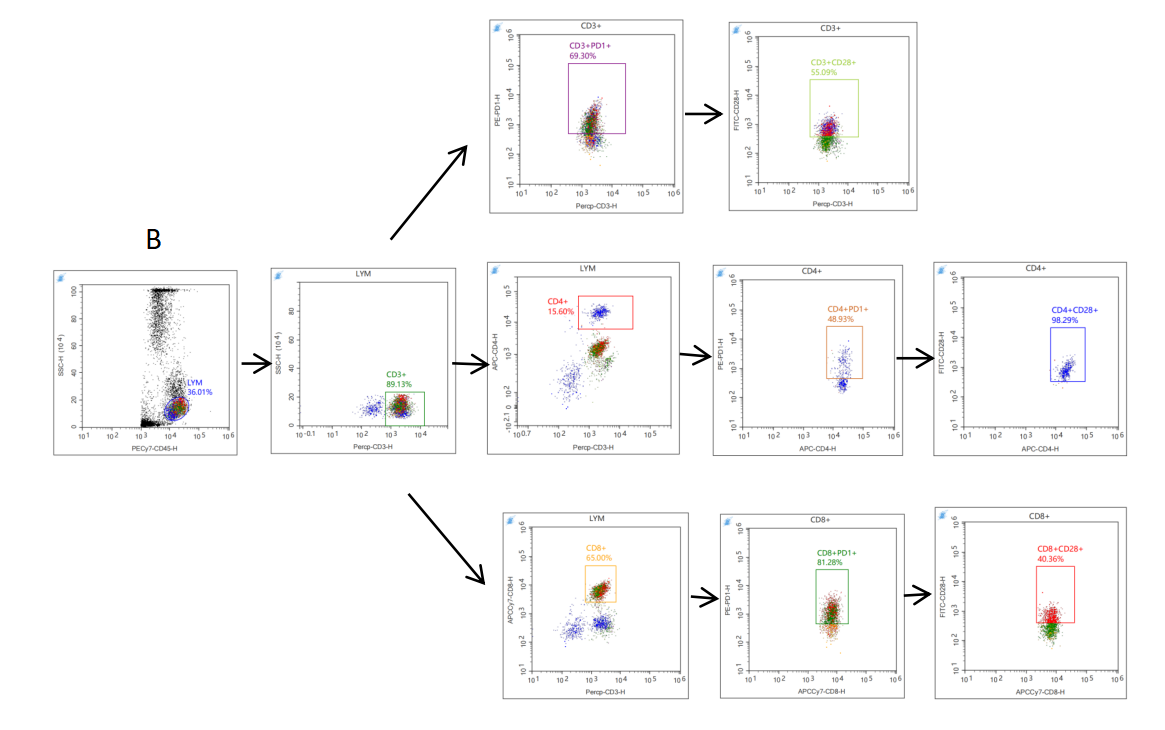


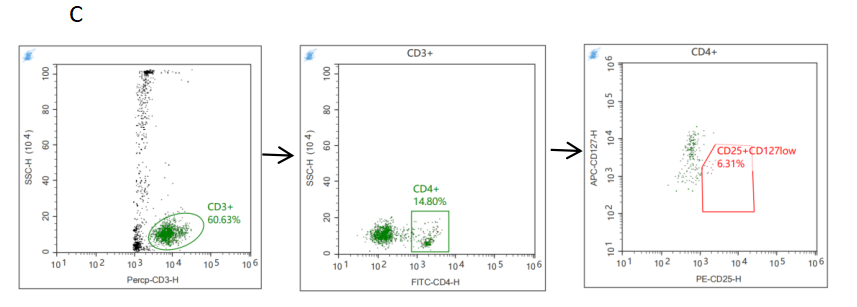


**Fig** Typical flow plots of the expression of granzyme, perforin, Tregs and other immune indicators. A, flow cytometry plots for granzyme and perforin detection; B, flow cytometry plots for PD-1 detection; C, flow cytometry plots for Tregs detection.
